# MRC chronic Dyspnea Scale: Relationships with cardiopulmonary exercise testing and 6-minute walk test in idiopathic pulmonary fibrosis patients: a prospective study

**DOI:** 10.1186/1471-2466-10-32

**Published:** 2010-05-28

**Authors:** Effrosyni D Manali, Panagiotis Lyberopoulos, Christina Triantafillidou, Likourgos F Kolilekas, Christina Sotiropoulou, Joseph Milic-Emili, Charis Roussos, Spyros A Papiris

**Affiliations:** 1 2nd Pulmonary Department, "Attikon" University Hospital, Athens Medical School, National and Kapodistrian University of Athens, Greece, 1 Rimini Street, 12462, Haidari, Greece; 2 Applied Biomedical Research & Training Center "Marianthi Simou", Department of Critical Care & Pulmonary Services, School of Medicine, National and Kapodistrian University of Athens, Ploutarchou 3, Str 10675 Greece; 3 Meakins-Cristie Laboratories, McGill University, 3626 St. Urbain Str, Montreal, Quebec H2X2P2, Canada

## Abstract

**Background:**

Exertional dyspnea is the most prominent and disabling feature in idiopathic pulmonary fibrosis (IPF). The Medical Research Chronic (MRC) chronic dyspnea score as well as physiological measurements obtained during cardiopulmonary exercise testing (CPET) and the 6-minute walk test (6MWT) are shown to provide information on the severity and survival of disease.

**Methods:**

We prospectively recruited IPF patients and examined the relationship between the MRC score and either CPET or 6MWT parameters known to reflect physiologic derangements limiting exercise capacity in IPF patients

**Results:**

Twenty-five patients with IPF were included in the study. Significant correlations were found between the MRC score and the distance (r = -.781, p < 0.001), the SPO_2 _at the initiation and the end (r = -.542, p = 0.005 and r = -.713, p < 0.001 respectively) and the desaturation index (r = .634, p = 0.001) for the 6MWT; the MRC score and *V*O_2 _peak/kg (r = -.731, p < 0.001), SPO_2 _at peak exercise (r = -. 682, p < 0.001), VE/VCO_2 _slope (r = .731, p < 0.001), VE/VCO_2 _at AT (r = .630, p = 0.002) and the Borg scale at peak exercise (r = .50, p = 0.01) for the CPET. In multiple logistic regression analysis, the only variable independently related to the MRC is the distance walked at the 6MWT.

**Conclusion:**

In this population of IPF patients a good correlation was found between the MRC chronic dyspnoea score and physiological parameters obtained during maximal and submaximal exercise testing known to reflect ventilatory impairment and exercise limitation as well as disease severity and survival. This finding is described for the first time in the literature in this group of patients as far as we know and could explain why a simple chronic dyspnea score provides reliable prognostic information on IPF.

## Background

In idiopathic pulmonary fibrosis (IPF), the combination of inflammatory and fibrotic lung parenchymal damage adversely affects lung mechanics and gas exchange and leads to progressive exertional breathlessness, the most prominent and disabling symptom in these patients [1-4]. Additionally, pulmonary vascular derangements both anatomic and functional [5,6] as well as myocardial disturbances [7,8] and peripheral muscle weakness related to the above changes [9,10] are all responsible for the progressive limitation in exercise capacity characteristically observed in these patients who usually have a more rapid an swallow breathing pattern [4,6,11-13].

Grading breathlessness with the aid of a variety of chronic dyspnea scales is practice in the assessment of IPF patients[14-17]. Recently, the Medical Research Council (MRC) chronic dyspnea scale has been used by several investigators including us, in the evaluation of disease severity in these patients[18,19]. The above mentioned studies have shown that the MRC score at the time of diagnosis may reflect severity and outcome[18-20]. Additionally, we have shown that that the MRC score significantly correlates with the CD_8+ _T lymphocytes in both tissue and bronchoalveolar lavage samples in these patients, subpopulation of lymphocytes that may be implicated in the pathogenesis of disease [21,22].

Previous studies have shown that physiological measurements obtained during cardiopulmonary exercise testing (CPET) [23] and the 6-minute walk test (6MWT) [24] two of the most commonly used exercise protocols may also provide information on the severity and outcome of IPF [25,26] in an even more useful and reliable way than resting physiological measurements such as pulmonary function testing values and arterial blood gases, in the prognostic evaluation of IPF patients [27-32]. More precisely, CPET provides an accurate assessment of abnormalities in the cardiovascular, respiratory, metabolic, peripheral muscle and neurosensory systems, under measured maximal physiological stress [23]. CPET parameters such as peak oxygen consumption (VO_2 _peak), oxygen pulse, ΔPa_O2_/ΔV_O2_, the ventilatory equivalent for carbon dioxide (VE/VCO_2_) at peak exercise and PaO_2 _slope are found to be significant predictors of survival in IPF patients [15,33,34]. Furthermore, parameters such as the distance walked and the level of desaturation during the 6MWT, a practical inexpensive simple walking test that examines submaximal levels of exertion, which might better reflect daily physical activities, are also found to be predictors of survival in this group of patients [27-29].

In an effort to better understand why a simple chronic dyspnea score, such as the MRC provides information on the severity and survival of IPF patients, we hypothesized that the MRC score could reflect physiologic derangements limiting exercise capacity in IPF patients. We tested our hypothesis by examining the potential relationship of the MRC score with well established and quantifiable parameters of either cardiopulmonary exercise testing or 6-minute walk test.

## Methods

### Subjects and Setting

This study was approved by the Institutional Ethics Committee of "Attikon" University Hospital, National and Kapodistrian University of Athens, Greece. Written informed consent was obtained from each patient. We prospectively recruited patients examined at the outpatient clinic over a period of one year. All patients fulfilled the criteria of the American Thoracic Society, European Respiratory Society and the American College of Chest Physicians for the diagnosis of IPF: Abnormal pulmonary function studies with evidence of restriction and impaired gas exchange, bibasilar reticular abnormalities with minimal ground glass opacities on high resolution computerized tomography scans, transbronchial lung biopsy or bronchoalveolar lavage showing no features to support an alternative diagnosis, age older than 50 years, insidious onset of otherwise unexplained dyspnea on exertion, duration of illness more than 3 months, bibasilar, inspiratory crackles (dry or "Velcro"-type in quality)[2,3]. Secondary causes of lung fibrosis were excluded: none of the patients had a history of environmental or occupational exposure, drug toxicity or autoimmune rheumatic disease, as documented by history, clinical and immunological tests. It is to be noted that the patients included in the present study are different new patients that have not been included in our previous studies [18,20-22].

### Dyspnea

Dyspnea was assessed at diagnosis by the treating physicians (EDM, PL, CT) using the modified MRC chronic dyspnea self-administered questionnaire consisting of six questions about perceived breathlessness: 0, no dyspnea; 1, slight dyspnea (shortness of breath when hurrying on the level or walking up a slight hill); 2, moderate dyspnea (walks slower than people of the same age on the level because of breathlessness); 3, moderately severe dyspnea (stops because of breathlessness when walking at own pace on the level); 4, severe dyspnea (stops for breath after walking about 100 yards or after a few minutes on the level); 5, very severe dyspnea (too breathless to leave the house or breathless when dressing or undressing)[18,19]. The Modified Borg Scale used at both the 6MWT and CPET is described as follows: 0, no breathlessness at all, 0.5 Very, very slight, just noticeable, 1 very slight, 2 Slight breathlessness, 3 moderate, 4 some what severe, 5 severe breathlessness, 6, 7 very severe breathlessness, 8, 9 very, very severe (almost maximum) and 10 maximum [16,17].

### Pulmonary Functional Tests (PFTs)

Lung function tests were done at diagnosis or at an interval not over passing 15 days at stable condition from the realization of the 6MWT and the CPET. PFTs included forced expiratory volume during the first second of expiration (FEV_1_), forced vital capacity (FVC), total lung capacity (TLC), and single-breath carbon monoxide diffusing capacity (DLCO) all measured by MasterScreen Body apparatus (Erich Jaeger GmbH, Wuerzburg, Germany) Measurements were expressed as both percent of predicted normal and as absolute values [35,36].

### The 6 Minute Walk Test

The 6MWT test was done according to the ATS guidelines [24]. The test was performed along a measured corridor in our Department. Participants were encouraged to cover as much distance as possible. The following data were collected and analyzed: distance (meters), duration (minutes), saturation at the initiation of the test, saturation at the end of the test, pulse at initiation and at the end of the test, the difference in saturation before and after the test, blood pressure systolic and diastolic before the test, Borg scale score before and after the test, age (years), height (meters) and body weight (kilograms)

### Cardiopulmonary Exercise Testing

The CPET was performed using a standardized protocol in accordance with the American Thoracic Society/American College of Chest Physicians (ATS/ACCP) statement [23]. Patients were encouraged to take their medication as usual. Patients taking beta blockers were specifically excluded. On arrival at the exercise suite, patients were connected to a 12-lead electrocardiogram (Cardio Card, Oxycon Pro). Oxygen saturation was measured by digital pulse oxymetry (AutoCorr BCI) and blood pressure by a sphygmomanometer. Calibration was done according to the instructions of the manufacturers. All patients underwent a maximal or symptom-limited cardiopulmonary exercise test with an electromagnetically braked cycle ergometer (Ergometrics 900, Erich Jaeger GmbH, Wurzburg, Germany) using a ramp protocol. All tests were monitored continuously with three leads, II, V1 and V5. The protocol included: 3 min of sitting rest, 3 min of unloaded cycling (at 60 revolutions per min. plus or minus 5 revolutions per min.), followed by a progressively increasing work rate (WR) in a ramp fashion and 3 min of recovery. The WR increment for each ramped exercise test was individualized on the basis of each patient's pretest activity level (range, 8 to 25 Watts/min). The duration of the test was symptom-limited. Cardiopulmonary data were collected and analyzed with an exercise metabolic unit (Oxycon Pro Erich Jaeger GmbH, Wurzburg, Germany). The following parameters were recorded: Heart rate (HR), minute ventilation (VE), tidal volume (VT), peak oxygen consumption (*V*O_2 _peak), peak oxygen consumption/Kg (*V*O_2 _peak/Kg), % VO_2 _predicted, VE/VCO2 slope, VE/VCO_2 _at AT, VT/IC, Borg scale at peak exercise, respiratory rate (RR), total ventilation (VE), oxygen pulse (O_2_P), oxygen saturation (SPO_2_), anaerobic threshold (AT), breathing reserve (BR), Heart rate recovery (HRR), Heart rate reserve (HRRes).

### Statistical analysis

Data are presented as mean ± standard deviation. (± SD). The non-parametric Spearman correlation coefficient was calculated to describe the relationships between variables. A p-value less than 0.05 was considered significant. Furthermore for variables that were significantly correlated with MRC, simple logistic regression models were fitted to identify predictors of MRC (separated in two categories, 0-2 and 3-4). Odds ratios (OR) and their 95% confidence intervals were calculated. Variables significant at 0.05 level were also entered in multiple stepwise logistic regression analysis to examine their independent effect on MRC groups. Statistical analyses were done using SPSS v.13.0.0 (Chicago, IL).

## Results

The population studied consisted of 25 patients with clinical and radiological features of IPF. The demographic and clinical characteristics of the study population at the time of IPF diagnosis are given in detail in Table 1. Seventeen patients were male with a mean age of 67.5 years (± 8.3). Only one patient was a current smoker, the rest being no smokers (44%) ex-smokers (52%) respectively. Two patients had an MRC score of 0, eight a score of 1, eight a score of 2, six a score of 3 and one a score of 4. No patient included in the study had a score of 5. The results of the PFTs are shown in Table 2. The study population had a restrictive pattern with a mean value for FEV_1_/FVC ratio, TLC and DLCO of 82.2% (± 4.5%), 61.4% (± 13.75%), and 45.6% (± 13.2%) respectively. Functional differences between non smokers and ex-smokers were examined and the only statistically significant difference between groups was found for FVC% (p = 0.03). Table 3 summarizes the results of the 6MWT while Table 4 the results of the CPET.

**Table 1:** Demographic and clinical data of the study population (n = 25)

Variables (n)	
Age, year (n = 25) (mean ± SD)	67.5 (± 8.3)
Sex (M/F) (n = 25)	17/8
Smoking history	
Ex smoker (n) %	13 (52%)
No smoker (n) %	11 (44%)
Smoker (n) %	1 (4%)
PY (mean ± SD)	25.4 (± 34.2)
MRC chronic dyspnea score	
0.0 (n) %	2 (8%)
1.0 (n) %	8 (32%)
2.0 (n) %	8 (32%)
3.0 (n) %	6 (24%)
4.0 (n) %	1 (4%)
5.0 (n) %	0 (0%)

M/F Male/Female, PY Pack/years

**Table 2:** Pulmonary Function Test Parameters of the study population (n = 25)

Parameter	n	Mean (± SD)
FEV_1 _(Liters)	25	1.95 ± .51
FEV_1_%	25	80.4 ± 18.8
FVC (Liters)	25	2.4 ± .6
FVC%	25	77.5 ± 21.8
TLC (Liters)	23	3.5 ± .85
TLC%	23	61.4 ± 13.7
DLCO (mmol/min/kPa)	23	3.6 ± 1.4
DLCO%	23	45.6 ± 13.2
FEV_1_/FVC	25	.82 ± .04

FEV_1 _Forced expiratory volume at 1 second, % per cent, FVC Forced vital capacity, TLC Total Lung Capacity, DLCO Diffusion capacity for carbon monoxide, FEV_1_/FVC: FEV_1_/FVC ratio

**Table 3:** Results of the 6 minute walk test (6MWT) in the study population (n = 25) in total as well as divided in groups based on the MRC score (0-1, 2, 3-4).

				MRC								
	**Total**			**0-1**			**2**			**3-4**		

	**N**	**Mean**	**SD**	**N**	**Mean**	**SD**	**N**	**Mean**	**SD**	**N**	**Mean**	**SD**

Distance	25	326.40	153.06	10	462.60	85.18	8	278.50	123.59	7	186.57	90.68

^1^SPO_2 _at the initiation %	25	94.84	2.23	10	96.60	1.51	8	93.63	1.51	7	93.71	2.29

^1^SPO_2 _at the end %	25	87.64	5.61	10	92.60	4.06	8	84.63	3.78	7	84.00	4.00

^2^DSPO_2_	25	7.20	4.29	10	4.00	3.89	8	9.00	3.55	7	9.71	2.75

PULSE at the initiation	25	80.16	13.75	10	76.00	13.46	8	83.00	17.21	7	82.86	9.53

PULSE at the end	24	104.25	17.15	9	105.89	18.59	8	102.75	19.89	7	103.86	14.09

^3^BP Systolic (mmHg)	25	123.60	12.71	10	128.00	8.88	8	119.38	14.74	7	122.14	14.68

^3^BP Diastolic (mmHg)	25	76.00	6.45	10	78.50	4.12	8	75.00	9.26	7	73.57	4.76

BORG scale at the initiation	25	0.18	0.35	10	0.10	0.32	8	0.19	.37	7	0.29	0.39

BORG scale at the end	25	2.02	1.75	10	1.20	0.89	8	2.38	1.77	7	2.79	2.34

All values are shown as mean ± standard deviation (SD)

^1 ^SPO_2 _oxygen saturation

^2 ^DSPO_2 _Difference of saturation between the end and the initiation of the test

^3 ^BP Blood pressure

**Table 4:** Results of the cardiopulmonary exercise testing (CPET) in the study population (n = 25) in total as well as divided in groups based on the MRC score (0-1, 2, 3-4).

				MRC								
	**Total**			**0-1**			**2**			**3-4**		

	**N**	**Mean**	**SD**	**N**	**Mean**	**SD**	**N**	**Mean**	**SD**	**N**	**Mean**	**SD**

VO_2 _peak (ml/min)	25	1233.28	437.61	10	1391.80	305.87	8	1307.75	573.62	7	921.71	281.54

VO_2 _peak (%)	24	73.38	19.11	9	83.89	15.02	8	70.00	20.71	7	63.71	17.56

VO_2 _peak/kg (ml/kg/min)	25	15.46	3.90	10	18.38	2.68	8	14.49	3.24	7	12.41	3.40

AT (ml/kg/min)	21	11.57	3.48	10	12.97	2.68	5	11.36	2.95	6	9.42	4.36

Oxygen pulse (ml/beat)	25	9.19	2.83	10	10.16	2.31	8	9.51	3.36	7	7.44	2.35

Oxygen pulse (%)	24	78.17	22.62	9	92.22	23.69	8	70.25	14.27	7	69.14	22.36

SPO_2 _peak	25	87.72	5.68	10	93.00	3.62	8	84.00	3.30	7	84.43	4.39

VE peak (L/min)	25	54.20	17.52	10	56.20	15.82	8	54.50	19.26	7	51.00	20.03

VE peak (%)	24	78.08	19.72	9	75.89	17.07	8	72.50	15.43	7	87.29	26.01

BR peak %	25	27.32	21.18	10	36.00	17.96	8	25.75	22.18	7	16.71	21.82

VE/VCO_2_	25	40.00	13.52	10	29.00	6.32	8	43.13	12.03	7	52.14	10.75

HR reserve	25	19.20	17.99	10	20.20	22.11	8	19.00	17.17	7	18.00	14.62

HR recovery	25	14.48	8.30	10	19.39	5.76	8	13.25	10.48	7	8.86	4.34

VE/VCO_2 _at AT	21	41.00	10.46	10	35.10	3.84	5	39.20	7.66	6	52.33	11.71

VT/IC	25	0.81	0.14	10	0.82	0.15	8	0.82	0.07	7	0.80	0.21

Borg scale at peak exercise	25	4.84	2.30	10	3.00	1.83	8	6.75	1.17	7	5.29	1.98

All values are shown as mean ± standard deviation (SD)

VO_2 _peak oxygen uptake at peak exercise, AT anaerobic threshold, SPO_2 _oxygen saturation, VE peak minute ventilation at peak exercise, VE/VCO_2 _slope: slope of relationship between VE and VCO_2_, HR Heart rate, VT tidal volume, IC Inspiratory capacity

Selectively, the mean distance walked was 326.4 m (± 153.06), with a SatO_2 _at rest of 94.8% (±2.2%) and a SPO_2 _at the end of the 6MWT of 87.6% (± 5.6%). The mean *V*O_2 _peak was 1233.3 ml/min (± 437.6), the *V*O_2 _peak/kg 15.5 ml/kg/min (± 3.9) and a % VO_2 _predicted 73.4% (± 19.1%). Four patients did not reach the anaerobic threshold, while the mean values for VE/VCO_2 _slope, VE/VCO_2 _at anaerobic threshold and VT/IC were 40 (± 13.5), 41 (± 10.46) and 0.81 (± 0.14) respectively. The mean saturation of oxygen at peak exercise was 87.7% (± 5.7%) and the mean Borg scale for dyspnea at peak exercise was 4.84 (± 2.3). Out of 25 patients, 16 patients (64%) stopped CPET due to mechanical constraint and the rest 9 (36%) due to leg discomfort.

To examine our hypothesis, we studied whether the MRC score at presentation was associated with any of the parameters of the 6MWT and CPET performed at maximum 15 days interval from the initial evaluation of the MRC at stable condition. The following correlations were found:

### Correlations between the MRC score and the parameters of the 6MWT

**Figure 1:**
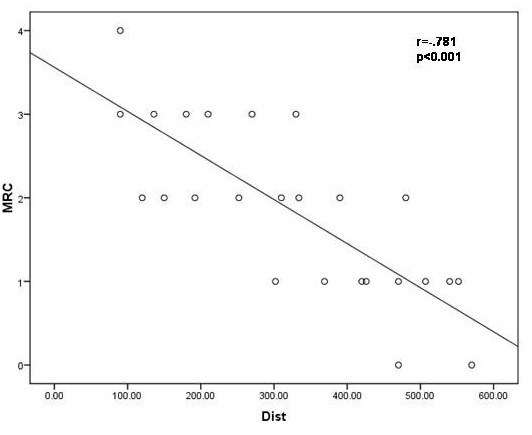
**The correlation between the MRC chronic dyspnea score and the distance in meters walked at the 6 minute walk test in the study population (n = 25) r = -.781, p < 0.001**.

Statistically significant correlations were found between the MRC score and the distance walked (r = -.781, p < 0.001), the SPO_2 _at the initiation and at the end of the test (r = -.542, p = 0.005 and r = -.713, p < 0.001 respectively) and the difference in saturation before and after the test (r = .0634, p = 0.001) [Table 5, Figure 1] Correlations between the MRC score and parameters of the 6MWT such as the pulse at the initiation and at the end of the test, the blood pressure, and the Borg dyspnea scale were not found to be statistically significant.(data not shown)

**Table 5:** Statistically significant relationships between the MRC chronic dyspnea score and the parameters of the 6 minute walk test (6MWT) in the study population quantified using Spearman's rank correlation coefficient

6MWT parameter		MRC score
Distance	r	**-.781**
	p	**<.001**
SPO_2 _at initiation	r	**-.542**
	p	**.005**
SPO_2 _at the end	r	**-.713**
	p	**<.001**
DSPO_2_	r	**0.634**
	p	**.001**

SPO_2 _oxygen saturation, DSPO_2 _difference of saturation between the end and the initiation of the test, BP Blood pressure

### Correlations between the MRC score and the parameters of the CPET

**Figure 2:**
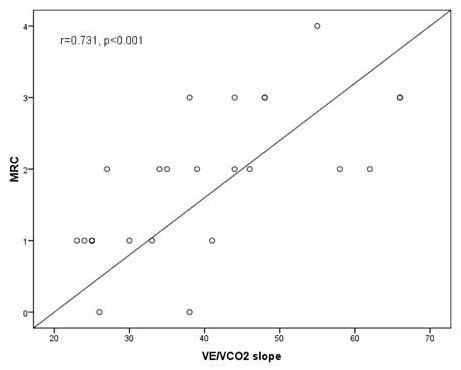
**The correlation between the MRC chronic dyspnea score and the VE/VCO_2 _slope at cardiopulmonary exercise test in the study population (n = 25) r = .731, p < 0.001**.

**Figure 3:**
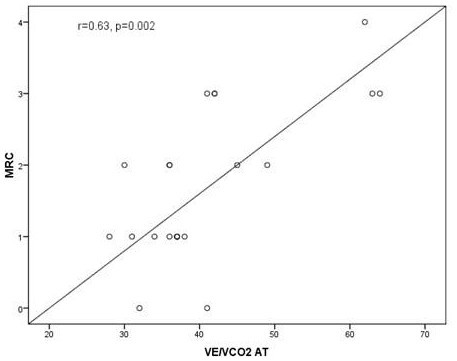
**The correlation between the MRC chronic dyspnea score and the VE/VCO_2 _at anaerobic threshold (AT) at cardiopulmonary exercise test in the study population (n = 25) r = .63, p < 0.002**.

Statistically significant correlations were found between the MRC score and the *V*O_2 _peak (r = -.496, p = 0.012), the VO_2 _peak % (r = -.481, p = 0.017), the *V*O_2 _peak/kg (r = -.731, p < 0.001), the saturation of oxygen at peak exercise (r = -.682, p < 0.001), the BR peak% (r = -.437, p = 0.029), the VE/VCO2 slope (r = .731, p < 0.001), the HR recovery (r = -.629, p = 0.001), the VE/VCO_2 _at AT (r = 0.630, p = 0.002) and the Borg scale at peak exercise (r = 0.5, p = 0.01) [Table 6, Figure 2, Figure 3]. Correlations between the MRC score and parameters of the CPET known to reflect restrictive mechanical constraint such as the VE peak and the VT/IC were not found to be statistically significant. (Data not shown)

**Table 6:** Statistically significant relationships between the MRC chronic dyspnea score and the parameters of the cardiopulmonary exercise testing (CPET) in the study population quantified using Spearman's rank correlation coefficient

CPET parameter		MRC score
VO_2 _peak	r	**-.496**
	p	**.012**
VO_2 _peak %	r	**-.481**
	p	**.017**
VO_2 _peak/kg	r	**-.731**
	p	**<.001**
SPO_2 _peak	r	**-.682**
	p	**<.001**
BR peak %	r	**-.437**
	p	**.029**
VE/VCO_2 _slope	r	**.731**
	p	**<0.001**
HR recovery	r	**-.629**
	p	**.001**
VE/VCO_2 _at AT*	r	**0.630**
	p	**.002**
Borg scale at peak exercise	r	**0.5**
	p	**.01**

VO_2 _peak: oxygen uptake at peak exercise, AT anaerobic threshold, SPO_2 _oxygen saturation, VE peak: minute ventilation at peak exercise, VE/VCO_2 _slope: slope of relationship between VE and VCO_2_, HR Heart rate, *VE/VCO_2 _at AT refers to 21 patients who reached AT

When applying simply logistic models for MRC categories [0-2 and 3-4] significant variables were found to be the FEV_1_, the FVC, the TLC, the distance walked, the *V*O_2 _peak, the VE/VCO_2 _slope and the VE/VCO_2 _at AT. While when applying a multiple stepwise logistic regression analysis with these independent variables, the only variable independently related to the MRC score with p < 0.05 is the distance walked at the 6MWT. (Data not shown)

## Discussion

In the present study we aimed to investigate for the first time in the literature as far as we know why a simple, clinical parameter such as the MRC chronic dyspnea score reported by the patient provides information on the severity and survival of IPF. We hypothesized that the MRC chronic dyspnea score mirrors one or multiple physiologic derangements leading to exercise limitation. Therefore the correlations between well established quantifiable features of CPET and the 6MWT with the MRC chronic dyspnea score at diagnosis were studied. According to our results, the MRC dyspnea score is significantly correlated with parameters such as the distance walked and the desaturation index of the 6MWT and with *V*O_2 _peak, the saturation of oxygen at peak exercise, the BR peak%, the VE/VCO_2 _slope, the VE/VCO_2 _at AT, the Borg scale at peak exercise and the HR recovery of the CPET. Among all parameters of the 6MWT and the CPET examined, the MRC score is shown to better and independently reflect the distance walked at the 6MWT. Furthermore, one third of the study population stopped exercise during CPET due to leg discomfort and not to mechanical constraint.

Dyspnea is a common and disabling symptom in IPF patients. Many studies have been performed to understand the pathophysiologic mechanisms underlying dyspnea in this group of patients in an effort to better and effectively face this problem which significantly limits functional capacity and quality of life. The MRC dyspnea score has been used to grade breathlessness in patients with IPF [18-22]. The present study shows that the MRC score is strongly related to the parameters of the 6MWT known to better predict severity and survival in IPF patients, such as the desaturation to ≤88% and the distance walked. We found that the higher the MRC score the shorter the distance walked and the lower the saturation of oxygen at the end of the test. Very few data exist on the literature concerning the relationship of the MRC score and the 6MWT in IPF. In the study of Mura et al. no correlation is found between the two, probably due to the fact that almost one third of the study population presented with both IPF and emphysema, in contrast to our patients which had no emphysema [37]. From a physiologic point of view our findings could be explained by the fact that desaturation during exercise in IPF patients reflects the overall impact of ventilation/perfusion mismatching, O_2 _diffusion limitation, low mixed venous PO_2 _and right to left intracardiac shunting in patients with a more rapid and swallow breathing pattern[6]. Walking in comparison to cycling recruits a wider range of muscle groups and results in the perception of dyspnea in disease states where oxygen delivery to peripheral muscles is limited. All the above mentioned derangements are mainly related to the inability of the IPF patient to increase lung volume and displace the thorax appropriately in the setting of an increased ventilatory drive, leading to a sense of breathing effort known as dyspnea and, as we speculate, recapitulated by the MRC score in the present study [6,10,38].

Among all parameters examined, the MRC score is shown to better and independently reflect the distance walked at the 6MWT. In the literature, this parameter is considered highly reproducible and reliable as a measurement compared to oxygen consumption and oxygen desaturation that are associated with significant variation in IPF patients [32]. Along with the desaturation of oxygen, the distance walked is a significant predictor of survival[27,29]. The distance walked is known to be related to patient effort, quadriceps strength and maximum inspiratory pressure in COPD patients [39]. In IPF patients, the distance walked is better correlated to functional parameters as well as to the alveolar-arterial gradient for oxygen[37]. In our study population significant correlation was not found between the MRC score and the Borg scale at the end of 6MWT, but a significant correlation was indeed found between the MRC score and the Borg scale at peak exercise during CPET. The Borg scale, a category scale with ratio properties, has been shown to be reproducible and responsive and ideal for the purpose of dyspnea assessment during CPET [6]. Such evidence does not exist for Borg Scale in 6MWT in IPF patients. In the literature it is shown that although desaturation is significantly more severe in IPF compared to COPD, dyspnoea assessed with the Borg scale is significantly more severe in COPD [17]. This could explain why this group of IPF patients with a significant desaturation during the 6MWT but a moderate average Borg scale walked a comparatively limited distance.

No study exists to our knowledge examining the relationship of the MRC score to CPET parameters in IPF. We found that the MRC score is significantly related to parameters reflecting ventilatory constraint such as the *V*O_2 _peak, the *V*O_2 _peak/Kg, the saturation of oxygen at peak exercise, the VE/VCO_2 _slope, VE/VCO_2 _at AT and the Borg scale at peak exercise. The peak oxygen consumption reflects the attainment of a limitation at some point in the oxygen conductance pathway from the lungs to the cytochrome-oxidase terminus of the electron transport chain. Thus physiologic derangements of IPF patients mentioned in the previous paragraph will be reflected in abnormally low values of this parameter, as well as of the saturation of oxygen at peak exercise [40,41]. Concerning the VE/VCO_2 _slope, during CPET a close linear relation exists between carbon dioxide and minute ventilation. This relation is more linear and less variable than that between oxygen and minute ventilation and the slope can be used to describe ventilatory response to exercise. In normal subjects the slope of VE/VCO_2 _reflects that 23-25 liters of VE is required to eliminate 1 liter of CO_2 _[21,40]. In our group of patients the mean value (± SD) for VE/VCO_2 _slope was found at 40 (± 13.5). An abnormal VE/VCO_2 _slope (greater than 34) during exercise has been suggested as an independent predictor of mortality in patients with chronic heart failure and often as a correlate for advanced disease[42]. In IPF patients, this parameter has not been extensively studied but a steeper slope is related to associated reduced cardiac output during exercise, increased pulmonary artery and capillary wedge pressures, increased dead space/total volume ratio and increased respiratory drive leading thus to more intense perception of dyspnea.

The prevalence of leg discomfort as the limiting factor in IPF is unknown but it is likely to be significant [6]. One third of our patients stopped exercise during CPET due to leg discomfort and not to mechanical constraint.

The present study includes a homogeneous population of IPF patients with demographic and functional characteristics comparable to previous studies examining 6MWT and CPET determinants of the MRC chronic dyspnea scale as a prognostic tool in IPF [18,20,27,28,32,43]. However, limitations of this study consist first in the relatively small number of patients and to the lack of IPF patients with MRC score of 5. However in patients with so high MRC scores, exercise is strongly limited leading to early completion of exercise tests before the limits of the pulmonary and cardiovascular systems are reached, which could negatively influence the results of the study [6]. Another limitation is the lack of information on the cardiac performance and pulmonary hypertension status of our patients, since data from heart ultrasounds are not included in the analysis, despite the increasing importance of the impact of pulmonary hypertension on gas exchange and exercise capacity in patients with pulmonary fibrosis[44].

## Conclusions

Based on the results of our study, the fact that the MRC score predicts severity and outcome of IPF, could be explained by the finding that the MRC score is related to major functional parameters of both maximal and submaximal exercise testing known to reflect ventilatory impairment, exercise limitation and extent of disease and found to be themselves highly prognostic of survival in IPF. This finding is for the first time described in the literature as far as we know and could aid in the understanding of exercise performance and limitation in this group of patients.

## Abbreviations

(AT): anaerobic threshold; (BR): breathing reserve; (CPET): cardiopulmonary exercise testing; (HR): Heart rate; (HRR): Heart rate recovery; (HRRes): Heart rate reserve; (IPF): Idiopathic pulmonary fibrosis; (MRC): Medical Research Chronic; (VE): minute ventilation; (6MWT): 6-minute walk test; (O_2_P): oxygen pulse; (SPO_2_): oxygen saturation; (PFTs): Pulmonary Functional Tests; (*V*O_2 _peak): peak oxygen consumption; (*V*O_2 _peak/Kg): peak oxygen consumption/Kg; (RR): respiratory rate; (VT): tidal volume; (VT/IC): tidal volume/inspiratory capacity; (VE): total ventilation.

## Competing interests

All authors declare that no financial or other potential competing interests exist with the study matter

The study has been partly financed by the "Kapodistrias" Research Program of the National and Kapodistrian University of Athens, Greece for the Academic Year 2008-2009

## Authors' contributions

EDM has contributed to the gathering and critical analysis of data and has written the manuscript. PL has conducted the cardiopulmonary exercise tests and has contributed to the interpretation of data. CT has conducted the 6MWTs, and contributed to the gathering and interpretation of all data. LFK has contributed to the critical analysis of all data and the drafting and critical review of the manuscript. CS has made the statistical analysis of data. JME and CR have contributed to the design of the study and the critical review of the manuscript. SAP has conceived of the study, has coordinated all authors and critically reviewed the data and the final version of the manuscript. All authors read and approved the final version of the manuscript.

## Pre-publication history

The pre-publication history for this paper can be accessed here:

http://www.biomedcentral.com/1471-2466/10/32/prepub
